# COVID-19 and the newly rediscovered multidisciplinarity

**DOI:** 10.2217/imt-2020-0205

**Published:** 2020-09-22

**Authors:** Melissa Bersanelli

**Affiliations:** ^1^Department of General and Specialistic Medicine, University Hospital of Parma, Medical Oncology Unit, Parma, Italy; ^2^Department of Medicine & Surgery, University of Parma, Parma, Italy

**Keywords:** anti-PD-1/PD-L1, COVID-19, immunosuppressed, immunotherapy, janus kinase inhibitors, pandemic, SARS-CoV-2, tocilizumab, virus

In the middle of the breathless fight against COVID-19, the eyes behind the facial masks of the physicians were those of either pneumologists, rheumatologists, oncologists, endocrinologists, surgeons and, in some cases, also dermatologists. This happened especially in centers heavily hurt by the SARS-CoV-2 outbreaks, involving all the hospital medical staff in the COVID-wards. Many of us have been uprooted from our quiet surgeries and day-hospitals to be catapulted into chilling emergency situations, never faced, nor imagined, during our previous professional routines. Each of us has tried to do our best, by applying a significant dose of resilience and taking hints from our original background, thus finally contributing to the imaginative therapeutic approaches rapidly emerging since the first few weeks of the pandemic.

Corroborated by this scenario, the present special focus issue collects the efforts from the most disparate branches of medicine, demonstrating the heterogeneity of approaches required by COVID-19 and contributing to opening our minds to a world of possibilities, from the bench to the bedside.

So it was that, taking lessons from rheumatology, several immunemodulators have been tested in the COVID-19 setting: Cala-García *et al.* described the efficacy of the anti-IL-6 tocilizumab against the SARS-CoV-2 infection [1], and Chang *et al.* reported the outcome of patients undergoing treatment with JAK inhibitors, providing suggestions from both rheumatology and dermatology [2]. Furthermore, taking the cue from cancer immunotherapy, anti-PD-1/PD-L1 immune-checkpoint inhibitors have been proposed as a possible solution to oppose the progression of the SARS-CoV-2 infection: along this line, this issue reports useful hints for research by Gatto *et al.* in their commentary [3]. On the other hand, oncological case series and representative cases developing COVID-19 during treatment with immune checkpoint inhibitors have been reported by Perrone *et al.* and by Rodrigo *et al.*, raising the issue of the risks from SARS-CoV-2 infection in special populations [4,5].

Again, from similarities between COVID-19 cytokine storm and the cytokine-release syndrome associated with CAR-T cell therapy in hematology, antibodies against IL-6 and GM-CSF have been employed by Melody *et al.* in an interesting case report [6].

Even coagulopathy experts have been involved in the treatment of this difficult disease, introducing low-weight molecular heparins among the therapeutic armamentarium, due to the likely significant contribution of the vascular compartment to the pathogenesis of the illness [7]. Finally, it seems that a thin but meaningful thread links COVID-19 pathogenesis and progression with metabolic diversities, in turn subtended by individual immunological patterns, as suggested in the present issue by Finelli *et al.* [8], constituting a crucial environment for the clinical evolution of this viral disease.

Unfortunately, SARS-CoV-2 infections often do not allow thoughtful reflections on the main components of the worsening condition of the patient, and the emergency frequently leads to messy therapeutic solutions. In the end, the same patient could have been treated with both immunesuppressants or immune-enhancers, with the aim of timely intercept of the upcoming phase of the disease.

If the cruciality of the immune response in COVID-19 pathogenesis is out of the question, it is still unclear if the morbidity could be more due to an initial immune-suppressive vulnerability or, on the contrary, to a late and aberrant immune hyperactivation.

The reports published in the present issue, aside from contributing to a valuable brainstorming on the underlying biological mechanisms to exploit for COVID-19 recovery, represent the testimony of the complicated rationale behind the current therapeutic approaches to the disease. If the use of tocilizumab still emerges as one of the most promising solutions, supported by scarce prospective evidence but pushed by the unmet need of an effective treatment [1,6], the attempt to outflank the immune system suppression has led to the proposal of an immuneprotection, exploiting physiological checkpoints to restore the immunocompetence [3]. It is the same rationale behind the immunotherapy of cancers, recently revolutionizing the therapeutic approach to solid tumors from an immunosuppressive perspective (e.g., chemotherapy) to an immunerestoring attempt [9]. The switch is difficult to accept, but in the case of oncology the clinical evidence strongly advocates the newest approach, likely the most physiological, relying more on the normalization than on the enhancement of the immunological status [10]. Indeed, the immune checkpoint blockade limits its effects in removing a block, allowing normal operation of the immune system to resume, more than enhancing the immune activation, that could even be harmful in the context of severe COVID-19.

Aside from the treatment of the SARS-CoV-2 infection, primary and secondary prevention is an incredibly important concern, especially in frail populations such as those affected by cancer, hematologic malignancies, obesity, pulmonary chronic obstructive disease, cardiovascular diseases or metabolic disorders [2–5,8]. The first tool to be exploited for prevention is knowledge: an accurate epidemiological framework of the disease in frail populations is unfortunately still missing, given the lack of the true denominator on which COVID-cases were reported. In the current scenario, the value of reliable predictions could be even more important in the lack of prophylactic solutions, especially in light of the fact that the only effective tool applied until today was social distancing. Reliable predictions of future outbreaks, of their setting and extension, would be the only tool to prevent the evolution of events in which the world has stumbled in the last few months.

**Figure 1.**
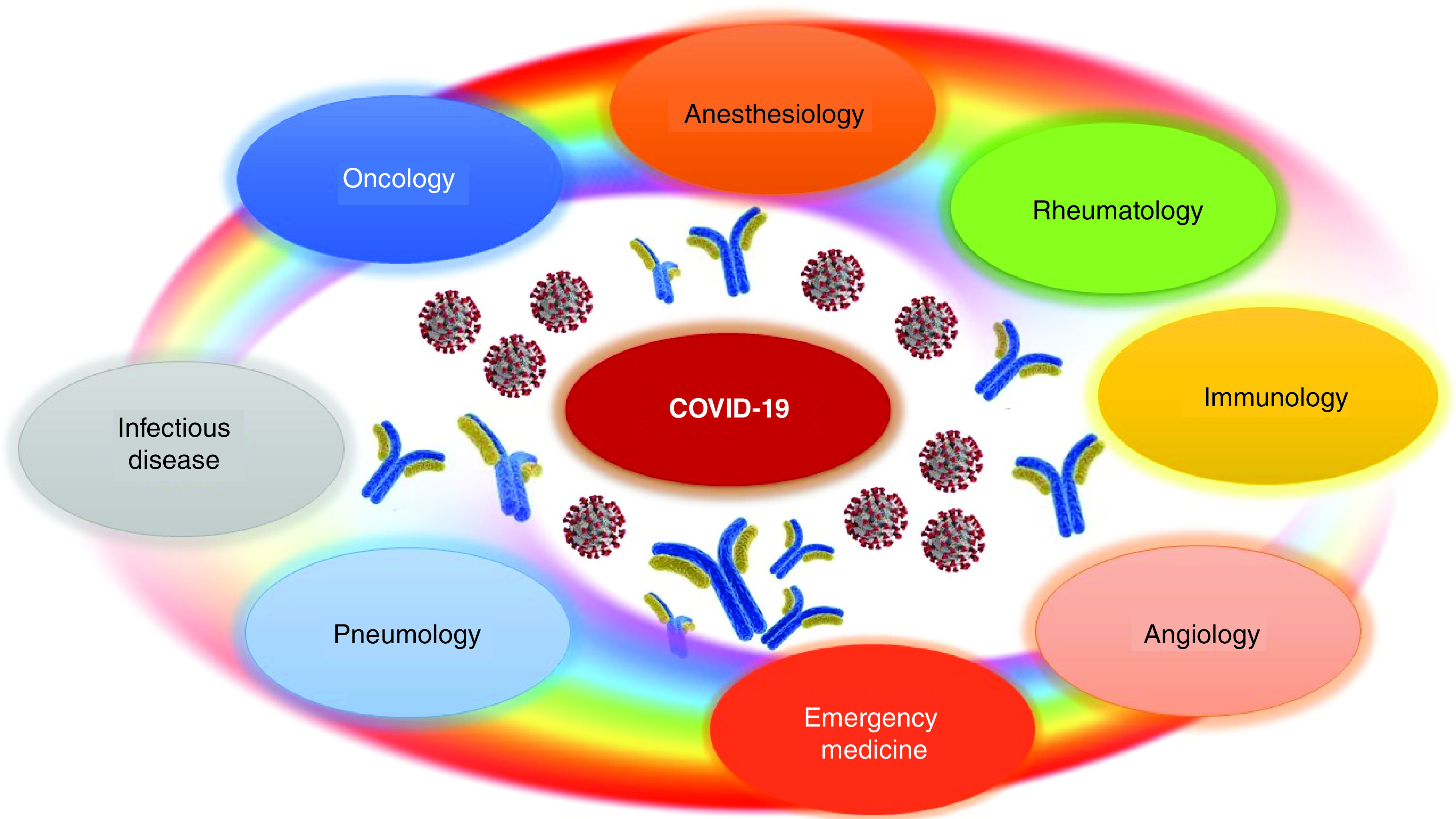
Multidisciplinarity in COVID-19 management.

In the meantime, the heartfelt research of therapeutic solutions remains a mission of physicians and researchers of all branches, sometimes contributing to creating new professional figures between immunology, infectious diseases and other specialties. Because union is strength (Figure 1).
